# Impact of frailty in patients with non‐valvular atrial fibrillation undergoing catheter ablation

**DOI:** 10.1002/joa3.13038

**Published:** 2024-04-09

**Authors:** Kyoko Soejima, Akihiko Nogami, Koichiro Kumagai, Kikuya Uno, Takashi Kurita, Itsuro Morishima, Fumiharu Miura, Ritsushi Kato, Tetsuya Kimura, Atsushi Takita, Masahiko Gosho, Kazutaka Aonuma, RYOUMA Investigators

**Affiliations:** ^1^ Department of Cardiology Kyorin University School of Medicine Mitaka Tokyo Japan; ^2^ Department of Cardiology, Faculty of Medicine University of Tsukuba Tsukuba Japan; ^3^ Heart Rhythm Center Fukuoka Sanno Hospital Fukuoka Japan; ^4^ Heart Rhythm Center Tokyo Heart Rhythm Hospital Tokyo Japan; ^5^ Division of Cardiovascular Center Kindai University School of Medicine Osaka‐Sayama Japan; ^6^ Department of Cardiology Ogaki Municipal Hospital Ogaki Japan; ^7^ Department of Cardiovascular Medicine Hiroshima Prefectural Hospital Hiroshima Japan; ^8^ Department of Arrhythmia Saitama Medical University International Medical Center Saitama Japan; ^9^ Primary Medical Science Department Daiichi Sankyo Co., Ltd. Tokyo Japan; ^10^ Data Intelligence Department Daiichi Sankyo Co., Ltd. Tokyo Japan; ^11^ Department of Biostatistics, Faculty of Medicine University of Tsukuba Tsukuba Japan

**Keywords:** cardiovascular, catheter ablation, elderly, frailty, non‐valvular atrial fibrillation

## Abstract

**Background:**

The relationships between frailty and clinical outcomes in elderly Japanese patients with non‐valvular atrial fibrillation (NVAF) after catheter ablation (CA) have not been established. We evaluated the frailty rate of patients undergoing CA for NVAF, examined whether CA for NVAF improves frailty, and analyzed the CA outcomes of patients with and without frailty.

**Methods:**

Elderly Japanese patients (≥65 years; mean age: 72.8 years) who participated in the real‐world ablation therapy with anti‐coagulants in management of atrial fibrillation registry and who responded to the frailty screening index survey were included (*n* = 213). Frailty and AF recurrence were assessed preoperatively and at 3 and 6 months after CA.

**Results:**

Twenty‐six patients (12.8%) were frail, 109 (53.7%) were pre‐frail, and 68 (33.5%) were robust. Cardiovascular (frailty: 0.5%/person‐year; pre‐frailty: 0.1%/person‐year; robust: 0.1%/person‐year) and cardiac (frailty: 0.5%/person‐year; pre‐frailty: 0.1%/person‐year; robust: 0.1%/person‐year) events, as well as major bleeding (frailty: 0.3%/person‐year; pre‐frailty: 0.1%/person‐year; robust: 0.1%/person‐year), were numerically more frequent in the frailty group. No deaths from cardiovascular or stroke/systemic thromboembolic events occurred. A large proportion of patients did not experience 3‐month (frailty: 96.2%; pre‐frailty: 96.3%; robust: 88.2%) or 6‐month (frailty: 88.5%; pre‐frailty: 91.7%; robust: 86.8%) AF recurrence after CA. Weight loss, walking speed, and fatigue improved in the frailty and pre‐frailty groups after CA.

**Conclusion:**

Japanese patients aged ≥65 years with frailty or pre‐frailty had improved frailty screening index components, such as weight loss, walking speed and fatigue, after CA. Therefore, elderly patients with frailty or pre‐frailty may benefit from CA for NVAF.

## Graphical abstract

Before catheter ablation, 12.8% of non‐valvular atrial fibrillation patients aged ≥65 years were classified as frail, 53.7% as pre‐frail, and 33.5% as robust by the 5‐item frailty screening index. The frailty and pre‐frailty groups showed a trend toward an improvement in weight loss, walking speed, and fatigue after catheter ablation.

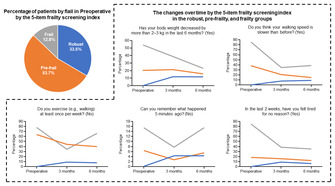

## 1 INTRODUCTION

Atrial fibrillation (AF) is the most common form of arrhythmia. The prevalence of AF increases with age,^1^ ranging from <1% in people aged <50 years to 10%–17% in people aged ≥80 years.^2^ The presence of AF can reduce quality of life and exercise tolerance,^3^, ^4^ and is associated with a 1.5‐ to 2‐fold increase in the risk of death and heart failure and a 5‐fold increase in the risk of stroke and systemic thromboembolic events (SEE).^3^, ^4^

Japan has the highest proportion of elderly people in the world,^5^, ^6^ so population aging is particularly relevant. Increased age is associated with an increased prevalence of frailty, with a recent study reporting that frailty affected 10% of adults aged 50–64 years and 43.7% of adults aged ≥65 years.^7^

Recent studies have demonstrated an association between frailty and AF.^8^, ^9^ Although the incidence of cardiovascular events, such as stroke and SEE, is high in patients with AF,^3^, ^4^ patients with AF complicated with frailty are at an even higher risk of stroke/SEE, major bleeding, and all‐cause death.^10^, ^11^

Catheter ablation (CA) is a well‐established treatment for AF,^12^, ^13^, ^14^ demonstrating superiority over antiarrhythmic drug therapy in patients with persistent or paroxysmal AF.^15^, ^16^ Previous studies have examined frailty and the effects of CA in patients with non‐valvular AF (NVAF).^17^, ^18^, ^19^ However, the prevalence of frailty and whether CA for NVAF improves frailty have not been fully examined in elderly Japanese patients with CA in the real‐world clinical setting.

This subgroup analysis of the Real‐world ablation therapY with anti‐cOagUlants in Management of Atrial fibrillation (RYOUMA) registry aimed to clarify the rate of frailty in patients undergoing CA for NVAF, assess whether CA for NVAF improves frailty in Japanese patients aged ≥65 years based on the 5‐item frailty screening index,^20^, ^21^ and describe CA outcomes and complications in patients with and without frailty. We also aimed to examine the rates of AF recurrence in the frailty, pre‐frailty, and robust groups.

## 2 METHODS

### 2.1 Study design

The RYOUMA registry (UMIN000026092) was a multicenter, prospective, observational study conducted between January 2017 and June 2018 by 77 investigators across 62 institutions in Japan. The detailed design of the RYOUMA registry has been described previously.^22^ The registry investigated the safety and efficacy of oral anticoagulant therapy during the periprocedural and long‐term follow‐up periods in Japanese patients undergoing CA for NVAF. The study protocol complied with the principles of the Declaration of Helsinki and local requirements for registries and ethical guidelines for clinical studies in Japan. Ethics approval was obtained from the institutional review boards of the participating institutions, and all patients provided written informed consent. All patients who were scheduled to undergo CA for the first time for NVAF were eligible for inclusion. Patients were not excluded for any baseline characteristics. The exclusion criteria were as follows: (1) informed consent not obtained from the patient or legal representative; (2) current or scheduled participation in a clinical trial involving pharmacotherapy; (3) previous AF ablation; and (4) ineligibility, as judged by the investigators.

### 2.2 Subgroup analysis population

Of the 3072 patients in the final RYOUMA registry analysis population, patients aged ≥65 years who provided written consent to be involved in this subgroup analysis and to undergo assessment using the 5‐item frailty screening index^20^, ^21^ at baseline were enrolled (Table 1). The participating sites were those that voluntarily agreed to participate, and the number of participating institutions was 12 (Table S1).

**TABLE 1:** Five‐item frailty screening index survey.^20^, ^21^

Item	Response/score
Has your body weight decreased by more than 2–3 kg in the last 6 months?	Yes = 1 point
Do you think your walking speed is slower than before?	Yes = 1 point
Do you exercise (e.g., walking) at least once per week?	No = 1 point
Can you remember what happened 5 min ago?	No = 1 point
In the last 2 weeks, have you felt tired for no reason?	Yes = 1 point

*Note*: A score of ≥3 indicates frailty, 1 or 2 indicates pre‐frailty, and 0 indicates robust.

### 2.3 Outcomes

Frailty assessments were made preoperatively, at 3 months after CA, and at 6 months after CA, and any improvement in frailty after CA was determined. The frailty status was assessed using the 5‐item frailty screening index.^20^, ^21^ Patients with a score of 0 points were classified as robust, patients with a score of 1–2 points were classified as pre‐frail, and patients with a score of ≥3 points were classified as frail. At the same time points, patients were also evaluated using electrocardiography and blood tests, and the attending physician determined whether AF had recurred at 3 or 6 months after surgery. Other variables, including left ventricular diastolic dimension, left ventricular systolic dimension, left ventricular ejection fraction, left atrial diameter, left atrial volume index, and left atrial volume, were measured using transthoracic echocardiography preoperatively and at 3 months after CA.

The event outcomes were the incidences of individual adverse events during the 1‐year observation period, including (1) stroke/SEE, (2) major bleeding (as defined by the International Society on Thrombosis and Haemostasis classification^23^), (3) intracranial bleeding, (4) cardiovascular events, (5) cardiac events, (6) all‐cause death, and (7) cardiovascular death.

### 2.4 Statistical methods

Categorical data are expressed as number (%) and were compared using the chi‐square test or Fisher's exact test, as appropriate. Continuous data are expressed as the median (interquartile range) and were compared using analysis of variance or the Kruskal–Wallis test, as appropriate. All statistical tests were two‐sided with a 5% significance level. All analyses were performed using SAS software, version 9.4 (SAS Institute, Cary, NC).

## 3 RESULTS

### 3.1 Patients' background characteristics

The background characteristics of the patients are shown in Table 2. Of the patients who underwent CA, 26 (12.8%) were classified as frail, 109 (53.7%) were classified as pre‐frail, and 68 (33.5%) were classified as robust. Of the robust, pre‐frailty, and frailty groups, patients in the frailty group median (75.5 [71.0–78.0] years) and had the lowest proportion of male patients (30.8%), the lowest systolic blood pressure, the highest diastolic blood pressure, the lowest body weight (56.8 [52.9–67.2] kg), the lowest creatinine clearance (61.2 [43.4–75.6] mL/min), and the highest CHA_2_DS_2_‐VASc score (4.0 [3.0–5.0]). The frailty group had the highest rate of warfarin use (11.5%) and the lowest rate of paroxysmal AF (46.2%). Of the 213 patients who underwent CA (radiofrequency: *n* = 124; cryoballoon: *n* = 54; hot balloon: *n* = 9; laser balloon: *n* = 2; combined: *n* = 23; other: *n* = 1), 4 experienced complications (cardiac tamponade: *n* = 1; puncture site bleeding: *n* = 1; hemorrhagic adverse event: *n* = 1; other: *n* = 1). There were no differences in the usage rate of a particular direct oral anticoagulant (DOAC) or DOAC dose among the three groups.

**TABLE 2:** Patients' background characteristics.

	Frailty *n* = 26	Pre‐frailty *n* = 109	Robust *n* = 68	*p* value*
Sex, male	8 (30.8)	69 (63.3)	48 (70.6)	0.0016
Age, years	75.5 (71.0–78.0)	72.0 (68.0–78.0)	71.5 (69.0–76.0)	0.122
Height, cm	151.5 (149.5–162.5)	163.0 (155.0–167.5)	164.8 (158.0–170.2)	<0.0001
Body weight, kg	56.8 (52.9–67.2)	61.2 (54.0–71.1)	63.2 (54.9–70.1)	0.45
BMI, kg/m^2^	24.55 (23.0–27.4)	23.3 (21.2–26.6)	22.95 (21.4–25.25)	0.142
SBP, mmHg	122.0 (114.0–132.0)	126.0 (113.0–137.5)	128.0 (119.0–144.0)	0.39
DBP, mmHg	81.0 (65.0–85.0)	76.0 (67.0–82.0)	77.5 (68.5–85.0)	0.44
CrCl, mL/min	61.2 (43.4–75.6)	61.7 (49.9–79.8)	67.9 (57.0–81.9)	0.199
CHA_2_DS_2_‐VASc score	4.0 (3.0–5.0)	3.0 (2.0–4.0)	3.0 (2.0–4.0)	0.014
CHA_2_DS_2_‐VASc score ≥3	23 (88.5)	76 (69.6)	44 (64.7)	0.196
HAS‐BLED score	3.0 (2.0–3.0)	3.0 (2.0–3.0)	2.5 (2.0–3.0)	0.73
HAS‐BLED score ≥3	15 (57.6)	59 (54.2)	34 (50.1)	0.45
Comorbidity				
Hypertension	21 (80.8)	78 (71.6)	49 (72.1)	0.63
Diabetes mellitus	7 (26.9)	27 (24.8)	17 (25.0)	0.97
Dyslipidemia	18 (69.2)	56 (51.4)	24 (35.3)	0.008
Heart disease	17 (65.4)	43 (39.4)	22 (31.4)	0.014
Kidney disease	6 (23.1)	19 (17.4)	4 (5.9)	0.04
Hepatic disorder	2 (7.7)	10 (9.2)	8 (11.8)	0.79
Cerebrovascular disease	3 (11.5)	23 (21.1)	6 (8.8)	0.08
Thromboembolism	1 (3.8)	6 (5.5)	3 (4.4)	0.91
Dementia	1 (3.8)	1 (0.9)	1 (1.5)	0.54
Cancer	4 (15.4)	18 (16.5)	9 (13.2)	0.84
AF type				0.21
Paroxysmal	12 (46.2)	74 (67.9)	40 (58.8)	
Persistent	11 (42.3)	23 (21.1)	21 (30.9)	
Long‐standing persistent	3 (11.5)	12 (11.0)	7 (10.3)	
Anticoagulants				0.31
Warfarin	3 (11.5)	9 (8.3)	2 (2.9)	
DOAC	23 (88.5)	100 (91.7)	65 (95.5)	
No OAC	0	0	1 (1.5)	
Type of DOAC				
Dabigatran	3 (11.5)	11 (10.1)	9 (13.2)	
Rivaroxaban	5 (19.2)	24 (22.0)	13 (19.1)	
Apixaban	9 (34.6)	23 (21.1)	20 (29.4)	
Edoxaban	6 (23.1)	42 (38.5)	23 (33.8)	
Dose of DOAC				
Off‐label high dose	3 (11.5)	7 (6.4)	0	
On‐label high dose	12 (46.2)	44 (40.4)	39 (57.4)	
On‐label low dose	6 (23.1)	33 (30.3)	19 (27.9)	
Off‐label low dose	2 (7.7)	16 (14.7)	7 (10.3)	

*Note*: Data are presented as median (interquartile range) or *n* (%).

Abbreviations: BMI, body mass index; CrCl, creatinine clearance; DBP, diastolic blood pressure; DOAC, direct oral anticoagulant; OAC, oral anticoagulant; SBP, systolic blood pressure.

* *p*‐values were calculated using the analysis of variance, Kruskal–Wallis test, chi‐square test, or Fisher's exact test.

The frailty group was older than the overall cohort (excluding patients aged <65 years and the Total subgroup). Differences between the frailty group and the overall cohort were observed for creatinine clearance, CHA_2_DS_2_‐VASc score, HAS‐BLED score, and comorbidities, and fewer patients in the frailty group had paroxysmal‐type AF. These differences suggest that the frailty group was higher risk than the overall cohort (data not shown).

### 3.2 Frailty assessment

Table 3 shows the number of patients who were classified as frail, pre‐frail, or robust preoperatively and at 3 and 6 months postoperatively. Of the 26 patients who were classed as frail preoperatively, 21 responded to the questionnaire at 6 months after CA. Of these, 2 (9.5%) were classed as robust and 10 (47.6%) were classed as pre‐frail, with only 9 (42.9%) still being classed as frail at 6 months after CA. Moreover, of the 109 patients who were classed as pre‐frail preoperatively, 86 responded to the questionnaire at 6 months after CA. Of these, 26 (30.2%) were classed as robust at 6 months after CA, and only 52 (60.5%) were still classed as pre‐frail. However, 8 patients (9.3%) who were initially classed as pre‐frail progressed to frailty at 6 months after CA.

**TABLE 3:** Number of patients in the frailty, pre‐frailty, and robust groups before catheter ablation (preoperative) and at 3 and 6 months after CA.

	Preoperative		
	Frailty *n* = 26	Pre‐frailty *n* = 109	Robust *n* = 68
3 months			
Frailty	8 (36.4)	13 (14.9)	2 (3.2)
Pre‐frailty	9 (40.9)	54 (62.1)	15 (24.2)
Robust	5 (22.7)	20 (23.0)	45 (72.6)
6 months			
Frailty	9 (42.9)	8 (9.3)	3 (5.4)
Pre‐frailty	10 (47.6)	52 (60.5)	13 (23.2)
Robust	2 (9.5)	26 (30.2)	40 (71.4)

*Note*: The percentage of patients in each group at 3 and 6 months was calculated based on the number of patients from the original preoperative population who responded to the questionnaires at 3 and 6 months (i.e., not all patients who were classified preoperatively responded to the questionnaires at 3 and 6 months).

**FIGURE 1:**
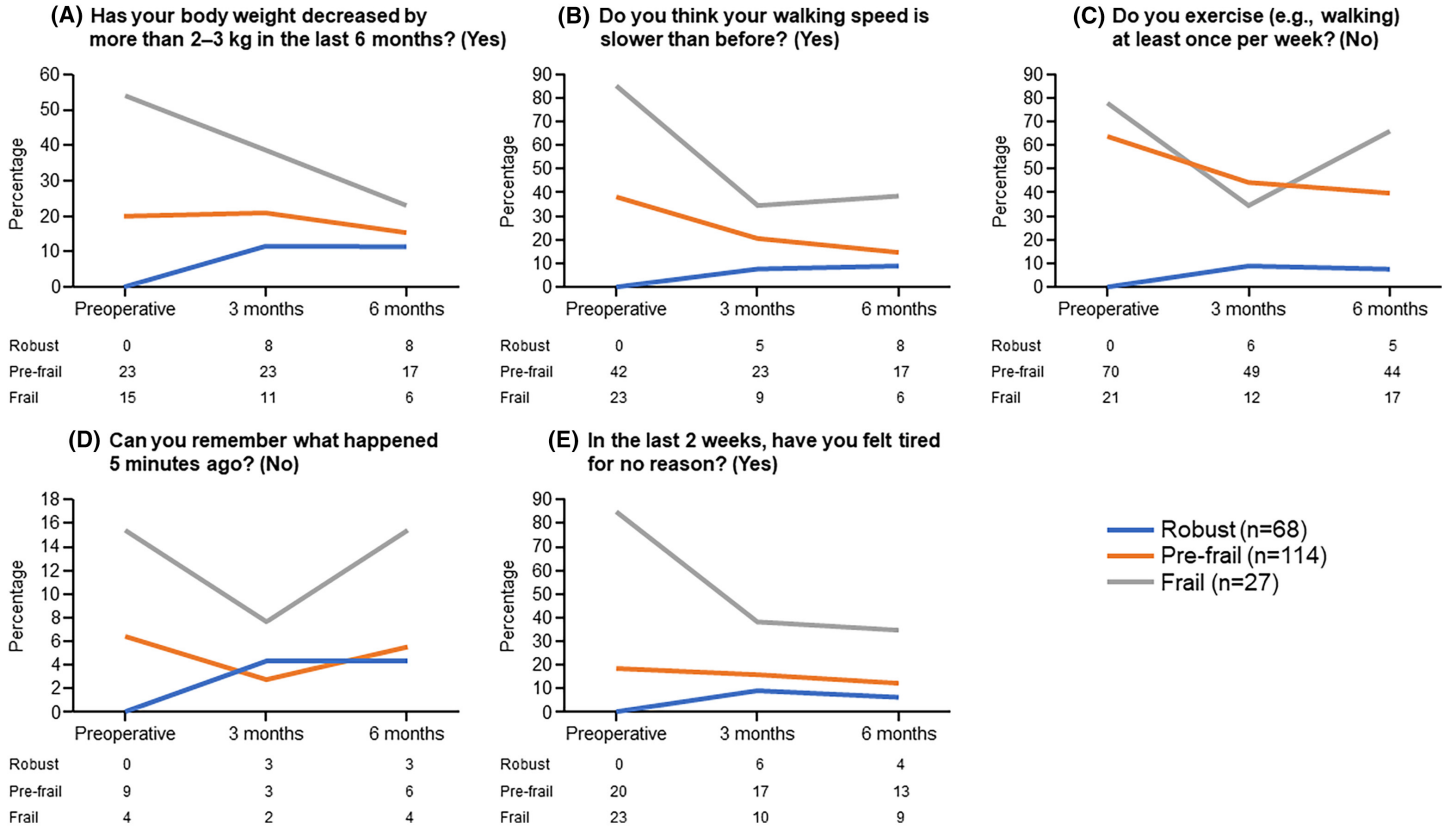
Changes in the five items of the frailty screening index before CA, 3 months after CA, and 6 months after CA in the robust, pre‐frailty, and frailty groups. CA, catheter ablation. A) Has your body weight decreased by more than 2–3 kg in the last 6 months? (yes). B) Do you think your walking speed is slower than before? (yes). (C) Do you exercise (e.g., walking). (D) Can you remember what happened 5 minutes ago? (no) at least once per week? (no). (E) In the last 2 weeks, have you felt tired for no reason? (yes)

The changes over time in the five frailty screening index items after CA in the robust, pre‐frailty, and frailty groups are shown in Figure 1. The frailty and pre‐frailty groups showed a trend toward an improvement in weight loss, walking speed, and fatigue after CA.

### 3.3 Event outcome assessment

The number (%/person‐year) of events for each outcome in the robust, pre‐frailty, and frailty groups after CA is shown in Table 4. The number of patients with major bleeding by frail status was 2 (0.3%/person‐year) in the frailty group, 7 (0.1%/person‐year) in the pre‐frailty group, and 4 (0.1%/person‐year) in the robust group. There was one case of cardiac tamponade in the robust group during the CA procedure, which was resolved by drainage. Cardiovascular events were observed in 3 patients (0.5%/person‐year) in the frailty group, 7 patients (0.1%/person‐year) in the pre‐frailty group, and 3 patients (0.1%/person‐year) in the robust group. Cardiac events were observed in 3 patients (0.5%/person‐year) in the frailty group, 6 patients (0.1%/person‐year) in the pre‐frailty group, and 3 patients (0.1%/person‐year) in the robust group. No deaths from cardiovascular events were observed in any of the three groups. Moreover, no cases of stroke/SEE or intracerebral hemorrhage were observed in any of the three groups.

**TABLE 4:** Event outcomes of patients in the robust, pre‐frailty, and frailty groups after catheter ablation.

	All *n* = 213	Frailty *n* = 26	Pre‐frailty *n* = 109	Robust *n* = 68
Stroke/SEE	0	0	0	0
Major bleeding	13 (0.0)	2 (0.3)	7 (0.1)	4 (0.1)
ICH	0	0	0	0
Cardiovascular events	14 (0.0)	3 (0.5)	7 (0.1)	3 (0.1)
Angina pectoris (exclude unstable angina) *n* (%)	5 (2.3)	2 (7.7)	3 (2.8)	0
Unstable angina *n* (%)	2 (0.9)	0	0	1 (1.5)
Heart Failure *n* (%)	5 (2.3)	1 (3.8)	3 (2.8)	1 (1.5)
Deep vein thrombosis *n* (%)	1 (0.5)	0	1 (0.9)	0
Cardiac tamponade *n* (%)	1 (0.5)	0	0	1 (1.5)
Cardiac events	13 (0.0)	3 (0.5)	6 (0.1)	3 (0.1)
All‐cause death	1 (0.0)	0	1 (0.0)	0
Cardiovascular death	0	0	0	0

*Note*: Data are presented as *n* (%/person‐year).

Abbreviations: ICH, intracerebral hemorrhage; SEE, systemic thromboembolic events.

### 3.4 AF recurrence and cardiac function after CA

**FIGURE 2:**
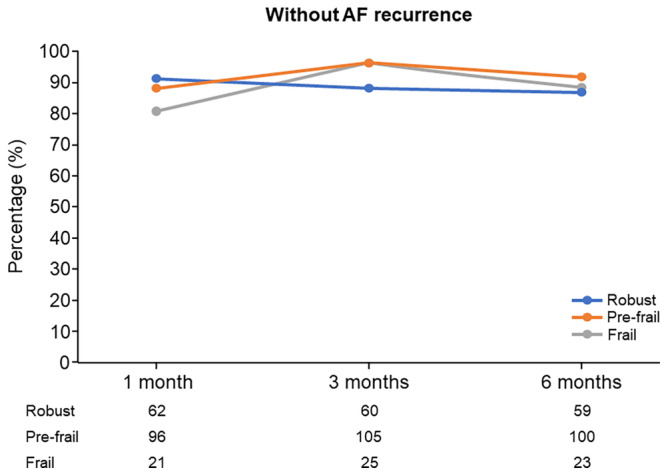
Percentage of patients without AF recurrence in the robust, pre‐frailty, and frailty groups at 1, 3, and 6 months after CA. AF, atrial fibrillation; CA, catheter ablation.

At 3 months after CA, the percentage of patients without AF recurrence was 96.2% in the frailty group, 96.3% in the pre‐frailty group, and 88.2% in the robust group. At 6 months after CA, the percentage of patients without AF recurrence was 88.5% in the frailty group, 91.7% in the pre‐frailty group, and 86.8% in the robust group (Figure 2). The rates of antiarrhythmic drug use at 1, 3, and 6 months after CA were 2 (2.7%), 0%, and 1 (3.8%) in the frailty group, 10 (10.1%), 2 (1.8%), and 3 (2.8%) in the pre‐frailty group, and 1 (1.5%), 2 (2.9%), and 3 (4.4%) in the robust group, respectively.

Numerically, left ventricular diastolic dimension, left ventricular systolic dimension, left ventricular ejection fraction, and left atrial diameter did not markedly change at 3 months after CA versus before CA in any of the three groups, but there was a trend toward an improvement in left atrial volume index at 3 months in all three groups (Table S2).

## 4 DISCUSSION

This is the first report to evaluate the improvement in the degree of frailty after CA for NVAF, assessed using the 5‐item frailty screening index.^20^, ^21^ The first finding was that CA showed a tendency to improve some components of the frailty screening index, such as weight loss, walking speed, and fatigue, in patients with frailty and pre‐frailty. The second finding was that the incidence of major bleeding, cardiovascular events, and cardiac events in patients with frailty was 0.3%/person‐year, 0.5%/person‐year, and 0.5%/person‐year, respectively, but no cardiovascular deaths were noted. Moreover, no cases of stroke/SEE or intracerebral hemorrhage were observed in any of the three groups. The third finding was that after CA, all three groups had low rates of AF recurrence.

Among the patients who underwent CA for NVAF, 12.8% were classified as frail, 53.7% as pre‐frail, and 33.5% as robust. In a recent report of elderly patients with NVAF in Japan, 19.5% were classified as frail, 62.6% were classified as pre‐frail, and 17.9% were classified as robust.^11^ In that study, the proportions of patients with frailty and pre‐frailty were higher than in this study, possibly because the previous study included subjects aged ≥75 years, whereas the present study included patients aged ≥65 years. This difference in the age distribution may have led to a difference in the proportion of patients with frailty or pre‐frailty. Moreover, the need for CA was determined at the attending physician's discretion; therefore, selection bias may have existed in patient enrollment, leading to the inclusion of more robust patients. This tendency to select more robust patients for CA has been noted previously in the consensus statement on CA for AF.^12^

This study showed that CA improved some components of the frailty screening index, including weight loss, walking speed, and fatigue. It is possible that an improvement in sinus rhythm restoration or a decrease in the AF burden after CA may have led to increased physical activity, in turn increasing appetite and limiting weight loss. It is also possible that discontinuation of antiarrhythmic drugs reduced weight loss, as antiarrhythmic drugs have been reported to produce gastrointestinal symptoms, reducing appetite. Moreover, the possibility of anorexia because of symptoms of right heart failure in patients with AF has been reported.^24^ Therefore, improving AF symptoms with CA may have led to the reduction in weight loss.

It is important to note that there is some overlap between AF symptoms and the features used to determine frailty (e.g., weight loss and shortness of breath). CA improved the symptoms of AF, which may have been reflected as an improvement in frailty. Another consideration is that more robust patients were selected in this study. These patients may be more energetic, influencing the improvement in physical activity and/or walking speed after CA. Patients with more severe frailty or very elderly patients might not demonstrate such an improvement in frailty after undergoing CA to treat AF; therefore, the results should be interpreted with caution in these groups.

Patients undergoing CA also receive lifestyle guidance, which may help them to become more health conscious, contributing to more comprehensive AF management.^25^ The improvement in walking speed in this study may be expected, as a previous study showed that CA for AF improves physical capability soon after the procedure.^26^ Thus, a higher proportion of patients after CA versus before CA answered “no” to “Do you think your walking speed is slower than before?,” in turn reducing the frailty screening index score and suggesting an improvement in walking speed. Fatigue may have been largely due to AF, explaining the improvement in fatigue after CA. Moreover, patients may have become more aware of lifestyle changes after CA, as shown in the AF Better Care (ABC) pathway (“A,” avoid stroke with anticoagulation; “B,” better symptom management; “C,” cardiovascular and comorbidity management, including lifestyle factors).^25^

Notably, a proportion of patients in the robust group were reclassified into the frailty and pre‐frailty groups during the postoperative follow‐up period. Although some items of the frailty screening index improved in the frailty and pre‐frailty groups, certain items did not improve in the robust group despite the efficacy of CA being similarly high in this group. As shown in Figure 1, the percentage of patients in the robust group with weight loss and memory impairment increased during the follow‐up period. One possible reason for weight loss is that as part of AF management, patients are instructed to exercise, eat healthy, and lose weight,^25^ and this would be particularly promoted for robust patients, possibly leading to greater weight loss and a consequent increase in frailty score.

In this study, the proportion of patients experiencing cardiovascular events and cardiac events was considered high after CA for NVAF in patients with frailty. This is in line with previous studies showing that frailty is a risk factor for major adverse cardiac events and cardiovascular mortality.^27^, ^28^ In addition to the high proportion patients with cardiovascular and cardiac events, the number of major bleeding events was high in patients with frailty who underwent CA for NVAF. The results are similar to those of the ANAFIE registry study, which showed that the rate of major bleeding was significantly higher in Japanese patients with NVAF complicated with frailty than in those classed as pre‐frail or robust,^11^ although no statistical comparisons among the three groups were made in the present study.

Patients with AF are at a high risk of stroke/SEE.^3^, ^4^ Although the incidence of stroke/transient ischemic attack in patients with AF undergoing CA is reported to be 0%–2.0%,^12^ this risk is thought to be further increased in those with frailty.^11^ However, no cases of stroke/SEE were observed in this study.

This study also demonstrated that both the frailty and pre‐frailty groups had low rates of AF recurrence after CA, as did the robust group. This may have been because of the large number of patients with paroxysmal AF in the pre‐frailty and frailty groups (*n* = 86) compared with the lower number of patients with long‐lasting (*n* = 34) or permanent (*n* = 15) AF in these groups. The low AF recurrence rates may also be attributable to the short follow‐up period of 6 months. Given that the rates of postoperative antiarrhythmic drug use were low, this is not likely to be the main reason for the low AF recurrence rates. It is important to note that the first 3 months after CA for AF are referred to as the 3‐month blanking period, during which time AF recurrence is thought to be quite common and does not necessarily indicate procedural failure.^4^, ^29^ For this reason, guidelines recommend that physicians judge the success of AF ablation no earlier than 6 months after the procedure.^4^, ^29^, ^30^ Thus, although a certain proportion of patients in all three groups demonstrated recurrence within 6 months, this was expected. Further follow‐up of these patients beyond the 6‐month period would be useful to determine whether the incidence of AF recurrence differs between the frailty/pre‐frailty groups and the robust group or whether the AF recurrence rates simply result from natural variation during the 6‐month post‐procedural period.

### 4.1 Limitations

This study has some limitations that should be noted. First, the short follow‐up period meant that long‐term outcomes were not assessed. The follow‐up period at facilities that perform CA is often 6 months, hence the follow‐up period of 6 months in the present study. Ideally, AF recurrence should also be assessed after the 6‐month post‐procedural period. Longer term outcomes should be assessed in the future to gain further insight into the safety of CA in patients with NVAF complicated with frailty. Second, the frailty screening index is a questionnaire administered by participants. Therefore, it remains unclear whether CA affects outcomes in frail patients who are more unwell, such as very elderly patients or patients with severe frailty. Moreover, frailty and pre‐frailty were determined using a simple index, the 5‐item frailty screening index, rather than comprehensive assessments. Third, patient enrollment was dependent on the clinical judgment of the physician and may have differed between facilities. Therefore, patients who were considered very frail may not have been included at the physicians' discretion, leading to possible selection bias. Fourth, the number of patients with frailty was only 26, which may have affected the reliability of the safety/efficacy data. Moreover, it is unknown whether the frailty status worsened in patients who did not complete the 5‐item frailty screening index at 3 and 6 months after CA. Therefore, further studies with larger sample sizes are needed in the future to confirm our findings. Fifth, this was not a controlled study, and there were no patients who did not undergo CA. Therefore, it is possible that CA reduced adverse outcomes in all groups, and this effect may have occurred independent of frailty. Sixth, statistical comparisons were not made between the frailty, pre‐frailty, and robust groups to identify significant differences between the groups in the frailty assessment, event outcome assessment, or AF recurrence and cardiac outcome results. Finally, the timing of frailty screening index completion and the cardiac function tests did not coincide.

## 5 CONCLUSIONS

Among elderly patients with NVAF who underwent CA, approximately 10% were classified as frail and 50% as pre‐frail. In this population, frailty screening index components, such as weight loss, walking speed, and fatigue, seemed to result from impaired physical activity, which suggests that elderly patients may benefit from CA for NVAF. However, large‐scale studies are needed in the future to confirm our findings.

## FUNDING INFORMATION

This work was supported by Daiichi Sankyo Co., Ltd. Daiichi Sankyo Co., Ltd. was involved in the study design, planning of the data analysis, data interpretation, and development of the manuscript, but was not involved in data management and statistical analysis.

## CONFLICT OF INTEREST STATEMENT

KS has received honoraria from Boehringer Ingelheim, Daiichi Sankyo, Abbott, Medtronic, and Johnson & Johnson. KK has received honoraria from Daiichi Sankyo, Boehringer Ingelheim, Fukuda Denshi, Nihon Kohden, Toray, and Japan Lifeline. KU has received honoraria from Japan Lifeline. T Kurita has received honoraria from Bayer, Boehringer Ingelheim, Daiichi Sankyo, Bristol‐Myers Squibb, Abbott, Medtronic, and Johnson & Johnson. AN has received honoraria from Boehringer Ingelheim, Daiichi Sankyo, Bristol Myers Squibb, Abbott, and Johnson & Johnson, and endowments from Medtronic and DVX. IM has received honoraria from Daiichi Sankyo and Abbott. FM has received honoraria from Medtronic, Abbott, and Biotronik. RK has received grant support from Boston Scientific, Abbott, Bayer, and Japan Lifeline. T Kimura and AT are employees of Daiichi Sankyo. MG has received honoraria from Pfizer, Ferring Pharma, Daiichi Sankyo, Merck Biopharma, and Novartis. KA has received honoraria from Boston Scientific, Japan Lifeline, Nihon Kohden, Biotronik, Toray Industries, ASTEC, Abbott, Boehringer Ingelheim, and Century Medical.

## ETHICS STATEMENT

Ethics approval was obtained from the institutional review boards of the participating institutions.

## PATIENT CONSENT STATEMENT

All patients provided written informed consent.

## Supporting information


Data S1.

## Data Availability

The deidentified participant data and the study protocol will be shared on a request basis for up to 36 months after the publication of this article. Researchers who make the request should include a methodologically sound proposal on how the data will be used; the proposal may be reviewed by the responsible personnel at Daiichi Sankyo Co. Ltd., and the data requestors will need to sign a data access agreement. Please directly contact the corresponding author to request data sharing.

## References

[1] Di Carlo A , Bellino L , Consoli D , Mori F , Zaninelli A , Baldereschi M , et al. Prevalence of atrial fibrillation in the Italian elderly population and projections from 2020 to 2060 for Italy and the European Union: the FAI project. Europace. 2019;21:1468–1475. 31131389 10.1093/europace/euz141

[2] Zoni‐Berisso M , Lercari F , Carazza T , Domenicucci S . Epidemiology of atrial fibrillation: European perspective. Clin Epidemiol. 2014;6:213–220. 24966695 10.2147/CLEP.S47385 PMC4064952

[3] Kirchhof P , Benussi S , Kotecha D , Ahlsson A , Atar D , Casadei B , et al. 2016 ESC guidelines for the management of atrial fibrillation developed in collaboration with EACTS. Eur J Cardiothorac Surg. 2016;50:e1–e88. 27663299 10.1093/ejcts/ezw313

[4] Calkins H , Kuck KH , Cappato R , Brugada J , Camm AJ , Chen SA , et al. 2012 HRS/EHRA/ECAS expert consensus statement on catheter and surgical ablation of atrial fibrillation: recommendations for patient selection, procedural techniques, patient management and follow‐up, definitions, endpoints, and research trial design. Europace. 2012;14:528–606. 22389422 10.1093/europace/eus027

[5] Voegele J . Rethinking silver: lessons from Japan's age‐ready cities. https://blogs.worldbank.org/voices/rethinking‐silver‐lessons‐japans‐age‐ready‐cities. Accessed August 28, 2023.

[6] Nakatani H . Population aging in Japan: policy transformation, sustainable development goals, universal health coverage, and social determinates of health. Glob Health Med. 2019;1:3–10. 33330747 10.35772/ghm.2019.01011 PMC7731274

[7] Fogg C , Fraser SDS , Roderick P , de Lusignan S , Clegg A , Brailsford S , et al. The dynamics of frailty development and progression in older adults in primary care in England (2006–2017): a retrospective cohort profile. BMC Geriatr. 2022;22:30. 34991479 10.1186/s12877-021-02684-y PMC8740419

[8] Villani ER , Tummolo AM , Palmer K , Gravina EM , Vetrano DL , Bernabei R , et al. Frailty and atrial fibrillation: a systematic review. Eur J Intern Med. 2018;56:33–38. 29936074 10.1016/j.ejim.2018.04.018

[9] Wilkinson C , Todd O , Clegg A , Gale CP , Hall M . Management of atrial fibrillation for older people with frailty: a systematic review and meta‐analysis. Age Ageing. 2019;48:196–203. 30445608 10.1093/ageing/afy180 PMC6424377

[10] Akishita M , Suzuki S , Inoue H , Akao M , Atarashi H , Ikeda T , et al. Frailty and outcomes in older adults with non‐valvular atrial fibrillation from the ANAFIE registry. Arch Gerontol Geriatr. 2022;101:104661. 35303601 10.1016/j.archger.2022.104661

[11] Akishita M , Suzuki S , Inoue H , Akao M , Atarashi H , Ikeda T , et al. Frailty screening index and atrial fibrillation outcomes in the All Nippon AF In the Elderly registry. Geriatr Gerontol Int. 2022;22:899–902. 35986506 10.1111/ggi.14458

[12] Calkins H , Hindricks G , Cappato R , Kim YH , Saad EB , Aguinaga L , et al. 2017 HRS/EHRA/ECAS/APHRS/SOLAECE expert consensus statement on catheter and surgical ablation of atrial fibrillation. Heart Rhythm. 2017;14:e275–e444. 28506916 10.1016/j.hrthm.2017.05.012 PMC6019327

[13] Nogami A , Kurita T , Kusano K , Goya M , Shoda M , Tada H , et al. JCS/JHRS 2021 guideline focused update on non‐pharmacotherapy of cardiac arrhythmias. Circ J. 2022;86:337–363. 34987141 10.1253/circj.CJ-21-0162

[14] Hindricks G , Potpara T , Dagres N , Arbelo E , Bax JJ , Blomström‐Lundqvist C , et al. 2020 ESC guidelines for the diagnosis and management of atrial fibrillation developed in collaboration with the European Association for Cardio‐Thoracic Surgery (EACTS): the Task Force for the diagnosis and management of atrial fibrillation of the European Society of Cardiology (ESC) developed with the special contribution of the European Heart Rhythm Association (EHRA) of the ESC. Eur Heart J. 2021;42:373–498. 32860505 10.1093/eurheartj/ehaa612

[15] Calkins H , Reynolds MR , Spector P , Sondhi M , Xu Y , Martin A , et al. Treatment of atrial fibrillation with antiarrhythmic drugs or radiofrequency ablation: two systematic literature reviews and meta‐analyses. Circ Arrhythm Electrophysiol. 2009;2:349–361. 19808490 10.1161/CIRCEP.108.824789

[16] Mark DB , Anstrom KJ , Sheng S , Piccini JP , Baloch KN , Monahan KH , et al. Effect of catheter ablation vs medical therapy on quality of life among patients with atrial fibrillation: the CABANA randomized clinical trial. JAMA. 2019;321:1275–1285. 30874716 10.1001/jama.2019.0692 PMC6450275

[17] Kundi H , Noseworthy PA , Valsdottir LR , Shen C , Yao X , Yeh RW , et al. Relation of frailty to outcomes after catheter ablation of atrial fibrillation. Am J Cardiol. 2020;125:1317–1323. 32147090 10.1016/j.amjcard.2020.01.049

[18] Yang P‐S , Sung J‐H , Kim D , Jang E , Yu HT , Kim TH , et al. Frailty and the effect of catheter ablation in the elderly population with atrial fibrillation—a real‐world analysis. Circ J. 2021;85:1305–1313. 33731545 10.1253/circj.CJ-20-1062

[19] Joung B , Yang PS , Sung JH , Jang E , Yu HT , Kim TH , et al. Catheter ablation can improve survival with the reduction of heart failure in frail patients with atrial fibrillation. Eur Heart J. 2020;41:ehaa946.0557.

[20] Yamada M , Arai H . Predictive value of frailty scores for healthy life expectancy in community‐dwelling older Japanese adults. J Am Med Dir Assoc. 2015;16(1002):e7–e11. 10.1016/j.jamda.2015.08.001 26385303

[21] Joint Committee for Comprehensive Risk Management Chart for the Prevention of Cerebro‐ and Cardiovascular Diseases . Comprehensive risk management chart for the prevention of cerebro‐ and cardiovascular diseases (in Japanese). J Jap Soc Intern Med. 2015;104:824–859. 10.2169/naika.104.824 26536749

[22] Nogami A , Soejima K , Morishima I , Hiroshima K , Kato R , Sakagami S , et al. Real‐world investigation on anticoagulation management before and after catheter ablation for atrial fibrillation in Japan. Circ J. 2022;87:50–62. 35989303 10.1253/circj.CJ-22-0290

[23] Schulman S , Kearon C , Subcommittee on Control of Anticoagulation of the Scientific and Standardization Committee of the International Society on Thrombosis and Haemostasis . Definition of major bleeding in clinical investigations of antihemostatic medicinal products in non‐surgical patients. J Thromb Haemost. 2005;3:692–694. 15842354 10.1111/j.1538-7836.2005.01204.x

[24] Gorter TM , van Melle JP , Rienstra M , Borlaug BA , Hummel YM , van Gelder IC , et al. Right heart dysfunction in heart failure with preserved ejection fraction: the impact of atrial fibrillation. J Card Fail. 2018;24:177–185. 29197548 10.1016/j.cardfail.2017.11.005

[25] Yang P‐S , Sung J‐H , Jang E , Yu HT , Kim TH , Lip GYH , et al. Applications of the simple atrial fibrillation better care pathway for integrated care management in frail patients with atrial fibrillation: a nationwide cohort study. J Arrhythm. 2020;36:668–677. 32782638 10.1002/joa3.12364 PMC7411200

[26] Yanagisawa S , Inden Y , Fujii A , Sakamoto Y , Tomomatsu T , Mamiya K , et al. Early improvement of daily physical activity after catheter ablation for atrial fibrillation in an accelerometer assessment: a prospective pilot study. Ann Noninvasive Electrocardiol. 2021;26:e12807. 32949223 10.1111/anec.12807 PMC7816803

[27] Damluji AA , Chung S‐E , Xue Q‐L , Hasan RK , Moscucci M , Forman DE , et al. Frailty and cardiovascular outcomes in the National Health and Aging Trends Study. Eur Heart J. 2021;42:3856–3865. 34324648 10.1093/eurheartj/ehab468 PMC8487013

[28] Quach J , Theou O , Godin J , Rockwood K , Kehler DS . The impact of cardiovascular health and frailty on mortality for males and females across the life course. BMC Med. 2022;20:394. 36357932 10.1186/s12916-022-02593-w PMC9650802

[29] January CT , Wann LS , Alpert JS , Calkins H , Cigarroa JE , Cleveland JC Jr , et al. 2014 AHA/ACC/HRS guideline for the management of patients with atrial fibrillation: a report of the American College of Cardiology/American Heart Association Task Force on Practice Guidelines and the Heart Rhythm Society. J Am Coll Cardiol. 2014;64:e1–e76. 24685669 10.1016/j.jacc.2014.03.022

[30] Wood KA , Barnes AH , Paul S , Hines KA , Jackson KP . Symptom challenges after atrial fibrillation ablation. Heart Lung. 2017;46:425–431. 28923248 10.1016/j.hrtlng.2017.08.007 PMC5811184

