# Supplementary material for: Impact of frailty in patients with non‐valvular atrial fibrillation undergoing catheter ablation
Source: J Arrhythm. 2024 Apr 9;40(3):463–71. doi: 10.1002/joa3.13038 (PMC11199813; doi:10.1002/joa3.13038)
Supplement: Supplementary file 1 — Data S1. [file JOA3-40-463-s001.docx]

**Supplementary Information**

**Supplementary Table 1.** Participating institutions in this subgroup analysis (n=12)

| Faculty of Medicine, University of Tsukuba | Akihiko Nogami |
| --- | --- |
| Fukuoka Sanno Hospital | Koichiro Kumagai |
| Hiroshima Prefectural Hospital | Fumiharu Miura |
| Kyorin University School of Medicine | Kyoko Soejima |
| Department of Medicine and Biosystemic Science, Kyushu University | Mitsuhiro Fukata |
| Makiminato Central Hospital | Satoshi Higa |
| Nagoya Daini Red Cross Hospital | Yukihiko Yoshida |
| Ogaki Municipal Hospital | Itsuro Morishima |
| Saitama Medical University International Medical Center | Ritsushi Kato |
| Tokushima Red Cross Hospital | Ryuji Otani |
| Tokushima University Hospital | Takeshi Soeki |
| Osaka Saiseikai Izuo Hospital | Yumie Matsui |

**Supplementary Table 2.** Cardiac function parameters before CA and 3 months after CA ablation in the robust, pre-frailty, and frailty groups

|  | | Preoperative | | 3 months | |
| --- | --- | --- | --- | --- | --- |
|  |  | n | Median (IQR Q1–Q3) | n | Median (IQR Q1–Q3) |
| LVDd, mm | Robust | 45 | 47.0 (44.5–50.0) | 40 | 46.4 (43.3–49.8) |
|  | Pre-frailty | 77 | 47.3 (42.7–51.0) | 80 | 47.4 (44.5–50.7) |
|  | Frailty | 21 | 46.9 (43.7–49.0) | 18 | 44.7 (42.6–48.2) |
| LVDs, mm | Robust | 44 | 30.5 (27.5–32.4) | 44 | 29.0 (27.7–31.9) |
|  | Pre-frailty | 77 | 29.2 (26.9–33.6) | 80 | 29.7 (27.1–32.2) |
|  | Frailty | 21 | 29.1 (26.6–30.8) | 18 | 27.7 (25.4–31.0) |
| LVEF, % | Robust | 45 | 66.0 (60.0–69.0) | 44 | 65.8 (62.0–68.0) |
|  | Pre-frailty | 78 | 64.9 (60.0–70.0) | 80 | 65.0 (62.0–69.0) |
|  | Frailty | 21 | 63.0 (59.0–66.0) | 18 | 66.7 (63.0–69.0) |
| LAD, mm | Robust | 45 | 40.4 (34.9–47.0) | 44 | 39.5 (36.0–44.1) |
|  | Pre-frailty | 76 | 40.3 (35.0–46.8) | 80 | 40.8 (36.3–46.1) |
|  | Frailty | 21 | 41.0 (38.0–46.5) | 18 | 39.2 (36.6–48.0) |
| LAV, mL | Robust | 28 | 72.5 (52.5–84.2) | 22 | 62.5 (49.5–72.0) |
|  | Pre-frailty | 43 | 60.0 (45.0–95.0) | 40 | 55.1 (45.5–68.5) |
|  | Frailty | 15 | 75.0 (50.0–89.3) | 12 | 53.5 (44.5–89.0) |
| LAVI, mL/m^2^ | Robust | 28 | 41.5 (35.0–54.5) | 22 | 37.0 (30.0–44.0) |
|  | Pre-frailty | 43 | 35.0 (27.0–54.0) | 40 | 32.5 (27.0–42.5) |
|  | Frailty | 15 | 42.0 (34.0–61.0) | 12 | 33.5 (27.5–55.5) |

Data are presented as median (IQR Q1–Q3).

Abbreviations: CA, catheter ablation; IQR, interquartile range; LAD, left atrial diameter; LAV, left atrial volume; LAVI, left atrial volume index; LVDd, left ventricular diastolic dimension; LVDs, left ventricular systolic dimension; LVEF, left ventricular ejection fraction.
